# NLRP3 Inflammasome Activation: A Therapeutic Target for Cerebral Ischemia–Reperfusion Injury

**DOI:** 10.3389/fnmol.2022.847440

**Published:** 2022-05-06

**Authors:** Lixia Wang, Wei Ren, Qingjuan Wu, Tianzhu Liu, Ying Wei, Jiru Ding, Chen Zhou, Houping Xu, Sijin Yang

**Affiliations:** ^1^Hospital of Chengdu University of Traditional Chinese Medicine, Chengdu, China; ^2^The Affiliated Traditional Chinese Medicine Hospital of Southwest Medical University, Luzhou, China; ^3^Guang'anmen Hospital, China Academy of Chinese Medical Sciences, Beijing, China; ^4^Preventive Treatment Center, The Affiliated Traditional Chinese Medicine Hospital of Southwest Medical University, Luzhou, China

**Keywords:** NLRP3 inflammasome activation, ischemic stroke, pyroptosis, cerebral I/R injury, mitochondrion

## Abstract

Millions of patients are suffering from ischemic stroke, it is urgent to figure out the pathogenesis of cerebral ischemia–reperfusion (I/R) injury in order to find an effective cure. After I/R injury, pro-inflammatory cytokines especially interleukin-1β (IL-1β) upregulates in ischemic brain cells, such as microglia and neuron. To ameliorate the inflammation after cerebral I/R injury, nucleotide-binding oligomerization domain (NOD), leucine-rich repeat (LRR), and pyrin domain-containing protein 3 (NLRP3) inflammasome is well-investigated. NLRP3 inflammasomes are complicated protein complexes that are activated by endogenous and exogenous danger signals to participate in the inflammatory response. The assembly and activation of the NLRP3 inflammasome lead to the caspase-1-dependent release of pro-inflammatory cytokines, such as interleukin (IL)-1β and IL-18. Furthermore, pyroptosis is a pro-inflammatory cell death that occurs in a dependent manner on NLRP3 inflammasomes after cerebral I/R injury. In this review, we summarized the assembly and activation of NLRP3 inflammasome; moreover, we also concluded the pivotal role of NLRP3 inflammasome and inhibitors, targeting the NLRP3 inflammasome in cerebral I/R injury.

## Introduction

**Figure 1:**
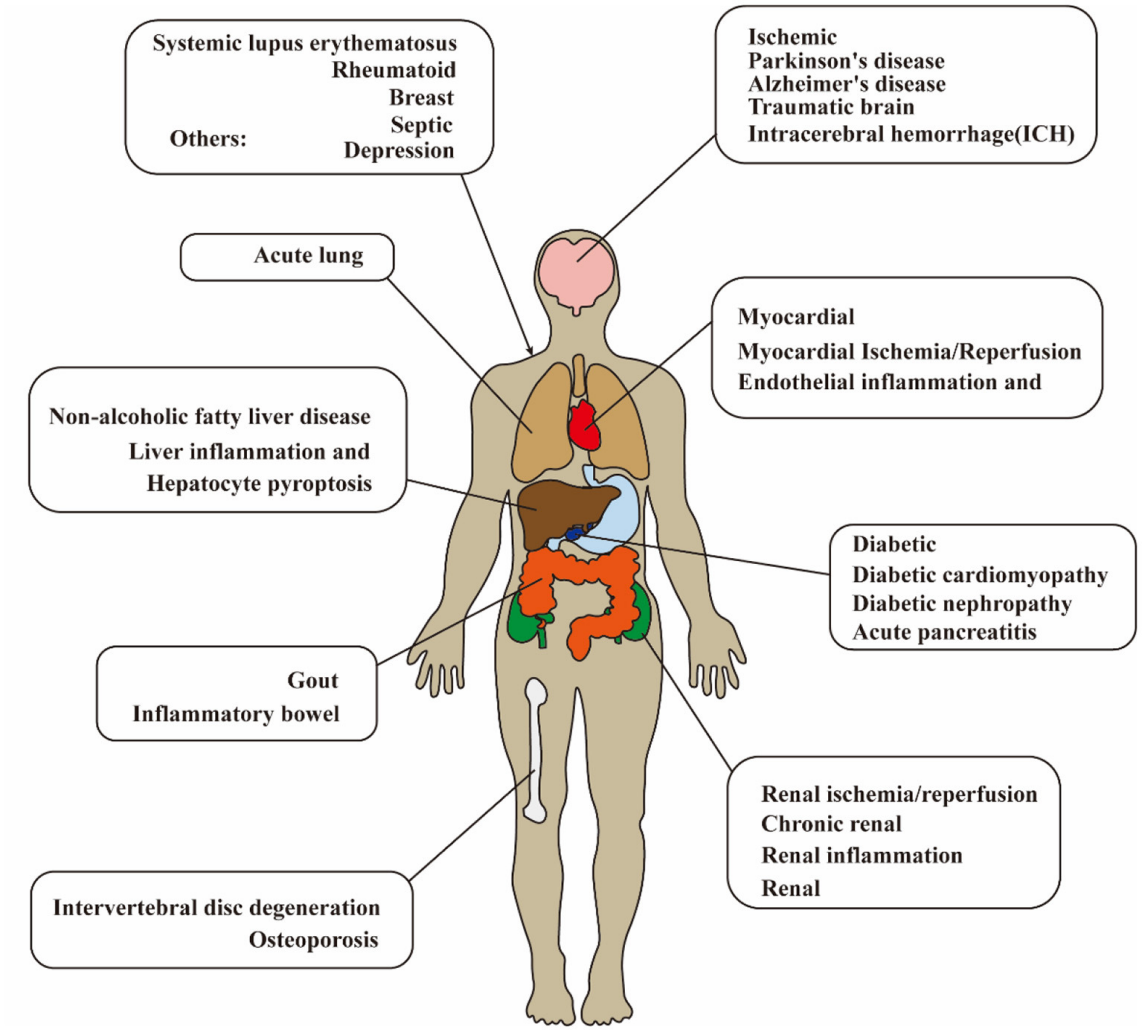
Diagram of NLRP3 inflammasome-related disease in human. The activation of NLRP3 inflammasome is extensively involved with the onset and progression of various diseases. NLRP3, pyrin domain-containing protein 3.

Ischemic stroke is the leading cause of disability in adults and has been a major health concern worldwide (Wang et al., 2017; Huang et al., 2022). Lack of understanding of the pathogenesis leads to limitations in the treatment of ischemic stroke. Currently, the immune response has shown both beneficial (Fernández-López et al., 2016) and detrimental effects (Jin et al., 2010) on the pathogenesis and prognosis of cerebral ischemia–reperfusion (I/R) injury. Oxidative stress, neuron death, and inflammation are involved in the pathogenesis of cerebral I/R injury (Pan et al., 2022a,b). Regarding neuroinflammation, the most definite mediator of inflammation after cerebral I/R injury is the cytokine interleukin-1 (IL-1) (Barrington et al., 2017). Nucleotide-binding oligomerization domain (NOD), leucine-rich repeat (LRR), and pyrin domain-containing protein 3 (NLRP3) inflammasome act as the upstream of IL-1, which regulates the mature and secretion of proinflammatory cytokines. After transient middle cerebral artery occlusion (MCAO) in mice, NLRP3 is the major contributor among the inflammasomes (Franke et al., 2021). It is confirmed that NLRP3 inflammasome has been a therapeutic target for cerebral I/R injury. As a new detective signaling platform, the NLRP3 inflammasome could be activated by a range of microbial infections, such as coronavirus (Zheng et al., 2020), *Staphylococcus aureus* (Mariathasan et al., 2006), and candida albicans hyphae (Joly et al., 2009). As shown in Figure 1 and Table 1, the activation of NLRP3 inflammasome is involved with the onset and progression of various diseases, and the research for medicines that inhibit the activation of NLRP3 inflammasome could be therapeutically beneficial. Therefore, numerous studies are pursuing to figure out the physiological structure, assembly, and activation of NLRP3 inflammasome and detect the potential pathogenic mechanisms.

**Table 1:** Diseases associated with pyrin domain-containing protein 3 (NLRP3) inflammasome.

**Disease**	**NLRP3 inflammasome inhibitor**	**Model**	**Animals and cells**	**Signaling**	**Effects**	**References**
Rheumatoid arthritis	MCC950	MCAO/R and OGD/R	Mice and SH-SY-5Y cells	The mitochondrial translocation of Drp1	Mitochondrial Function, ER Stress	Guo et al., 2018a
Endothelial inflammation and atherosclerosis	NLRP3 shRNA	Diabetes mellitus model	Diabetes patients and diabetic ApoE^−/−^ mice and human umbilical vein endothelial cells (HUVECs).	NLRP3 inflammasome signaling	Endothelial inflammation	Wan et al., 2019
Inflammatory bowel diseases	Glyburide	IL-10 mice	C57BL/6 mice and Patients with diagnosis of CD	NLRP3 inflammasome signaling	Inflammation	Liu et al., 2017
Renal fibrosis	MCC950	Renal fibrosis model	C57BL/6 mice	NLRP3 inflammasome signaling	Oxidative stress, inflammation, renal dysfunction,histological injury, and interstitial fibrosis	Li et al., 2019
Renal ischemia/reperfusion injury	Hydroxychloroquine	Renal I/R injury model	C57BL/6J mice and HK-2 cells	NF-κB signaling	Renal inflammation	Tang et al., 2018
Renal inflammation	B-cell lymphoma 6 (BCL6)	Spontaneously hypertensive rats (SHR) and Inflammation models	SHR, Wistar-Kyoto rats (WKY), and HK-2 cell	NLRP3 transcription	Inflammation in the renal cortex	Chen et al., 2017
Chronic renal dysfunction	Phloretin	Hyperuricemia model	C57BL/6 male mice and HK-2 cell	NLRP3 pathway	Inflammation	Cui et al., 2020
Traumatic brain injury (TBI)	NIMA-related kinase 7 (NEK7)-shRNA	Controlled Cortical Impact (CCI) Model	C57BL/6 mice and Primary Cortical Neurons	NEK7–NLRP3 signaling	Neuroinflammation and pyroptosis	Chen et al., 2019b
Acute pancreatitis (AP)	INF-39	Severe acute pancreatitis (SAP) Model	NLRP3^−/−^ C57BL/6 mice	NLRP3 inflammasome signaling	Inflammatory cascade and neutrophil infiltration	Fu et al., 2018
Systemic lupus erythematosus (SLE)	Methylprednisolone	SLE patients	–	NEK7-NLRP3 inflammasome signaling pathway	Inflammation	Ma et al., 2018
Ischemic stroke	Ketogenic Diet	MCAO Model and OGD/R	C57BL/6 mice and SH-SY-5Y cells	Mitochondrial translocation of Drp1	ER stress, apoptosis and inflammation	Guo et al., 2018b
Breast cancer	MiRNA-233-3p	Breast cancer cell lines	HMEC, MDA-MB231, MCF-7, and SKBR3 cell lines	MiR-233/NLRP3 inflammasome pathway	Immunoactivation and the growth of breast cancer	Zhang L. P. et al., 2019
Myocardial infarction	Colchicine	Myocardial Infarction Mouse Model	C57BL/6J mice	NLRP3 inflammasome signaling	Acute Inflammation	Fujisue et al., 2017
Diabetic cardiomyopathy	Empagliflozin	Diabetic db/db mice	Mice	sGC-cGMP-PKG pathway	Cardiomyocyte pyroptosis	Xue et al., 2019
Myocardial Ischemia/Reperfusion (I/R) Injury	BAY11-7082/MCC950	Myocardial ischemia/reperfusion (MI/R) injury and H/R Injury model	Sprague-Dawley rats and H9C2 Cell	NLRP3 inflammasome signaling	Pyroptotic cell death	Qiu et al., 2017
Diabetic nephropathy (DN)	Optineurin	High glucose culture	DN patient and Murine primary renal tubular epithelial cells (RTECs)	Mitophagy	Mitochondrial dysfunction	Chen et al., 2019a
Diabetic retinopathy	Fenofibrate	Diabetes model	C57BL/6 mice	Nrf2 signaling	Retinal leukostasis and vascular leakage	Liu Q. P. et al., 2018
Parkinson's disease (PD)	FTY720	PD model	C57BL/6J mice, BV-2 microglial, and SH-SY5Y neuroblastoma cell	PI3K/AKT/GSK-3β signaling pathway	Neuronal damage and microglia activation	Yao et al., 2019
Alzheimer's disease (AD)	MCC950	APPswe/PS1dE9 mice	Mice and microglia	NLRP3 inflammasome signaling	Amyloid accumulation	Dempsey et al., 2017
Intracerebral hemorrhage (ICH)	Adiponectin	ICH model (the injection of autologous blood)	Sprague-Dawley rats	NLRP3 inflammasome signaling	Inflammation	Wang S. H. et al., 2020
Septic shock	Cardamonin, MCC950	Injection of LPS	C57BL/6 mice and Bone-marrow-derived macrophages (BMDMs)	NLRP3 inflammasome signaling	Inflammation	Wang et al., 2019
Osteoporosis (OP)	Irisin	OP model (ovariectomy)	Sprague-Dawley rats	Nrf2 signaling	Apoptosis and inflammation	Xu et al., 2020
Non-alcoholic fatty liver disease (NAFLD)	Naringenin	Methionine-choline deficient (MCD) diet and cellular steatosis model with LPS and oleic acid (OA)	C57BL/6 mice, Primary hepatocytes, KCs, and HepG2 cells	NLRP3/NF-κB pathway	Inflammatory activation and lipid deposition	Wang Q. Y. et al., 2020
Liver inflammation and fibrosis	MCC950, IL-1 receptor antagonist (anakinra)	Atherogenic diet-fed foz/foz model and Methionine and choline deficient diet model	Mice, bone marrow-derived macrophages, primary hepatocytes, Kupffer cells	NLRP3 inflammasome signaling	Hepatocyte pyroptosis, liver inflammation and fibrosis	Wree et al., 2014; Mridha et al., 2017
Intervertebral disc degeneration (IVDD)	Melatonin	Human IVDs and AF puncture surgery for rats	Rats and nucleus pulposus (NP)	IL-1β/NF-κB-NLRP3 inflammasome positive feedback loop	Inflammatory response	Chen et al., 2020
Depression	Fluoxetine	Chronic mild stress model	C57BL/6 mice and primary macrophage/microglia	ROS- double-stranded RNA-dependent protein kinase (PKR)-NLRP3 Signaling Pathway	Inflammatory response	Du et al., 2016
Acute lung injury	Glybenclamide	Acute lung injury model induced by administering paraquat (PQ)	Sprague–Dawley rats	NLRP3-ASC-caspase-1 pathway	Inflammatory injury	Liu et al., 2015
Gout	β-hydroxybutyrate(BHB), Ketogenic Diet	Gout Model and Peritonitis Model	Human and C57BL/6 mice	NLRP3 inflammasome signaling	Inflammatory response	Goldberg et al., 2017

## The Assembly of NLRP3 Inflammasome

Inflammasome was first described by Fabio Martinon in 2002, which was the identification of a caspase-activating complex that consisted of a sensor (PRRs), an adaptor (ASC), and an effector (caspase1) (Martinon et al., 2002; Swanson et al., 2019). The PRRs are expressed in innate immune system cells (such as macrophages and neutrophils cells) (Abderrazak et al., 2015), and PRRs sense pathogen-associated molecular patterns (PAMPs) or damage/danger-associated molecular patterns (DAMPs) (Matzinger, 2002; Karasawa and Takahashi, 2017) for the innate immune system to defend against these “danger signals.” PRRs can be divided into many subgroups, including members of Toll-like receptors (TLRs), C-type lectin receptors (CLRs), NOD-like receptors (NLRs), and absent-in-melanoma 2 receptors (ALRs) (Lamkanfi and Dixit, 2014; Kelley et al., 2019). NLRP3 inflammasome is the most well-characterized inflammasome and closely related to the cleavage of caspase-1 (Martinon et al., 2002). NLRP3 is composed of central NOD, a C-terminal LRR domain, and an N-terminal pyrin domain (PYD) (Menu and Vince, 2011; Alishahi et al., 2019). ASC (apoptosis speck protein) has a C-terminal pyrin domain (PYD) and a C-terminal caspase recruitment domain (CARD) (Alishahi et al., 2019). The CARD in ASC is homotypic with the CARD in pro-caspase-1. After the LRR domain is sensitized by stimuli, NLRP3 self-oligomerizes through the interaction of homotype NODs (Alishahi et al., 2019), then, the N-terminal PYD domain in oligomerized NLRP3 facilitates homotypic PYD–PYD interactions between NLRP and adapter protein ASC (Liepinsh et al., 2003), and assembled ASC recruits pro-caspase-1 *via* C-terminal CARD–CARD interactions (Srinivasula et al., 2002). The assembly and activation of NLRP3 inflammasome promote the cleavage of pro-caspase-1 to form active caspase-1, which leads to the maturation and cleaves of proinflammatory cytokines, such as IL-1β and IL-18.

## The Activation of NLRP3 Inflammasome Under I/R Injury

**Figure 2:**
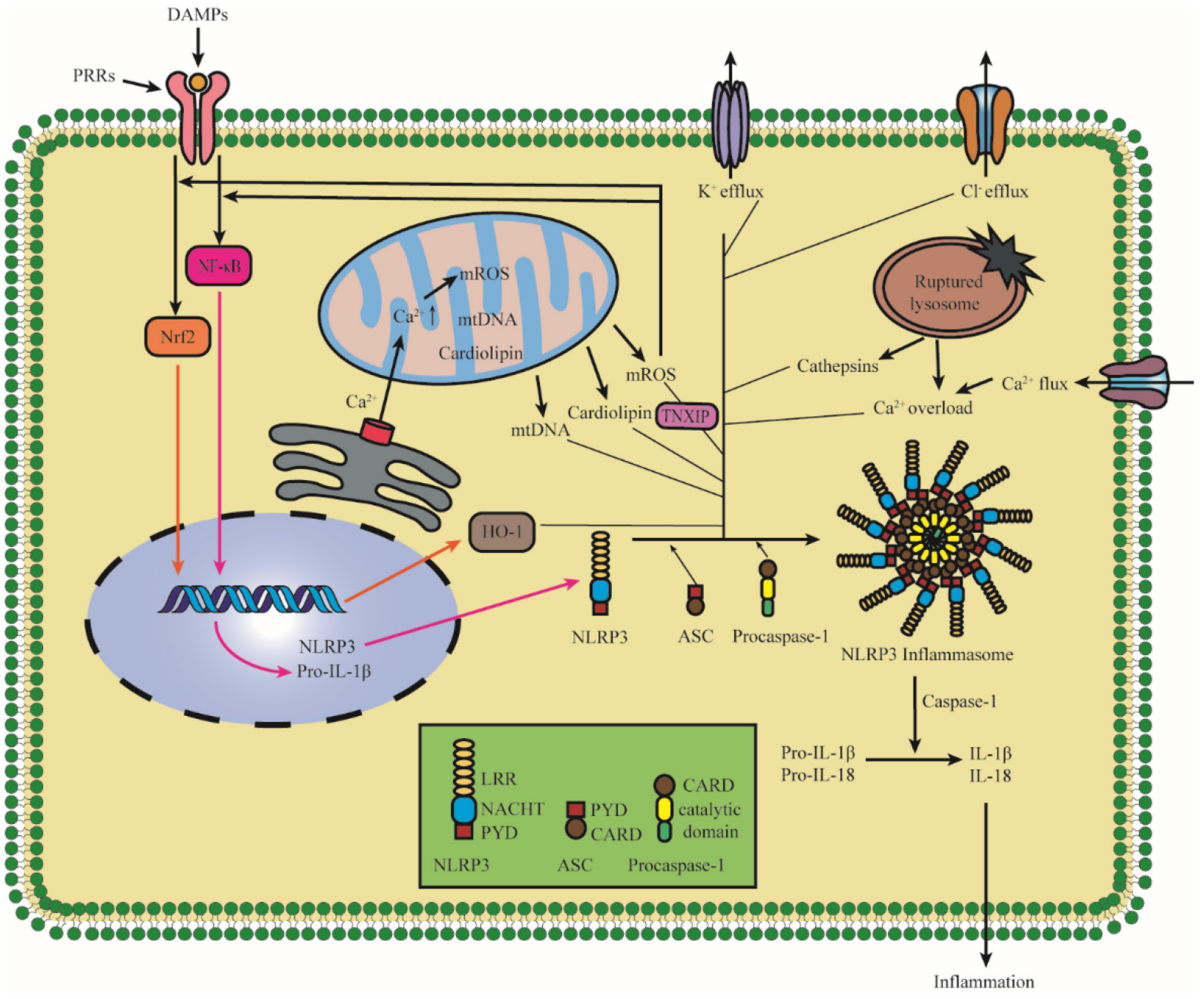
Diagram of NLRP3 inflammasome activation. The priming induces NLRP3 and pro-IL-1β upregulation in an NF-κB-dependent manner through translocating to the nucleus. The upstream DAMP and PAMP signaling events lead to the oligomerization of NLRP3 and the assembly of ASC and pro-caspase-1 to form NLRP3 inflammasome. There are mainly three models, which activate the activation of NLRP3 inflammasome: K^+^ efflux, ROS, and lysosomal damage, and specifically it also includes Cl^−^ efflux, Ca^2+^ overload, mtDNA, cardiolipin, and the nuclear translocation of Nrf2. The assembly and activation of the NLRP3 inflammasome lead to the cleavage of Caspase-1, which promotes the maturation and cleaves proinflammatory cytokines IL-1β and IL-18. NLRP3, pyrin domain-containing protein 3; NF-κB, nuclear factor-k-gene binding; HO-1, Hemeoxygenase-1; TNXIP, thioredoxin interacting protein; PRR, pattern recognition receptor; DAMPs, damage/danger-associated molecular patterns; Nrf2, NF-E2-related factor 2; ROS, reactive oxygen species; mtDNA, mitochondrial DNA.

Notably, the activation of NLRP3 inflammasome has been detected after I/R injury. It is confirmed that NLRP3 was upregulated significantly in 4 h after hypoxic-ischemic in rats, and the elevated levels of IL-1β were detected in 8 h after hypoxic-ischemic injury (Li et al., 2021b). A study of patients with acute ischemic stroke admitted <24 h showed that the level of serum concentration of NLRP3 was related to the increased risk of malignant brain edema (MBE) (Wang et al., 2021e). Therefore, elucidating the mechanism of NLRP3 activation and intervening early after the onset of ischemic stroke are of great importance for the treatment and prognosis of ischemic stroke. It is widely accepted that NLRP3 inflammasome activation is related to two signals: in signal I (priming), cytokines or PAMPs could lead to pro-IL-1β and NLRP3 upregulation in a nuclear factor-κ-gene binding (NF-κB)-dependent manner in response to the activation of proinflammatory cytokine receptors or transcription-modulating PRRs; in signal II (activation), various upstream DAMP and PAMP signaling events lead to the oligomerization of NLRP3 and the assembly of ASC and pro-caspase-1 to form NLRP3 inflammasome (Shao et al., 2015; Liu, Q. Y et al., 2018; Sho and Xu, 2019; Swanson et al., 2019). Recent studies demonstrate that there are mainly three models which activate the signal II in the activation of NLRP3 inflammasome, namely, ionic flux, mitochondrial destabilization, and lysosomal damage (Gong et al., 2018a; Kelley et al., 2019). All these models are shown in Figure 2. In this study, we described the activation of NLRP3 inflammasome after cerebral I/R injury.

### Ionic Flux

Intracellular ionic flux which includes K^+^ efflux, Ca^2+^ mobilization, and Cl^−^ efflux acts as an upstream regulation role in the activation of NLRP3 inflammasome (Gong et al., 2018a). K^+^ efflux is the most investigated target, the main reason for the decrease in intracellular K^+^ concentration is related to K^+^ channel opening (Di et al., 2018), membrane remodeling (Gianfrancesco et al., 2019), and membrane permeabilization change (Franchi et al., 2014); at the same time, many stimuli can trigger K^+^ effluxes such as adenosine triphosphate (ATP), nigericin, and monosodium urate (MSU) crystals (Nomura et al., 2015). The activation of NLRP3 could be blocked by inhibiting K^+^ efflux; therefore, the low intracellular K^+^ concentration is a key trigger for NLRP3 inflammasome activation (Pétrilli et al., 2007). It has demonstrated that K^+^-ATP channel pore-forming subunit Kir6.1 is a bona fide negative regulator of the NLRP3 inflammasome, and the suppression of Kir6.1 increases the accumulation of damaged mitochondria and production of reactive oxygen species (ROS) (Du et al., 2019; Hu et al., 2019). K^+^ efflux is a well-accepted upstream regulating signal for NLRP3 inflammasome, but some studies have reported that other signals could also affect NLRP3 inflammasome activation in a K^+^ efflux-independent manner. Imiquimod affects ROS production by regulating the quinone oxidoreductases, namely, NQO2 and mitochondrial Complex I to participate in the activation of the NLRP3 inflammasome, and the activation of the NLRP3 inflammasome by imiquimod is K^+^ efflux-independent (Groß et al., 2016).

### Mitochondrial Destabilization

**Figure 3:**
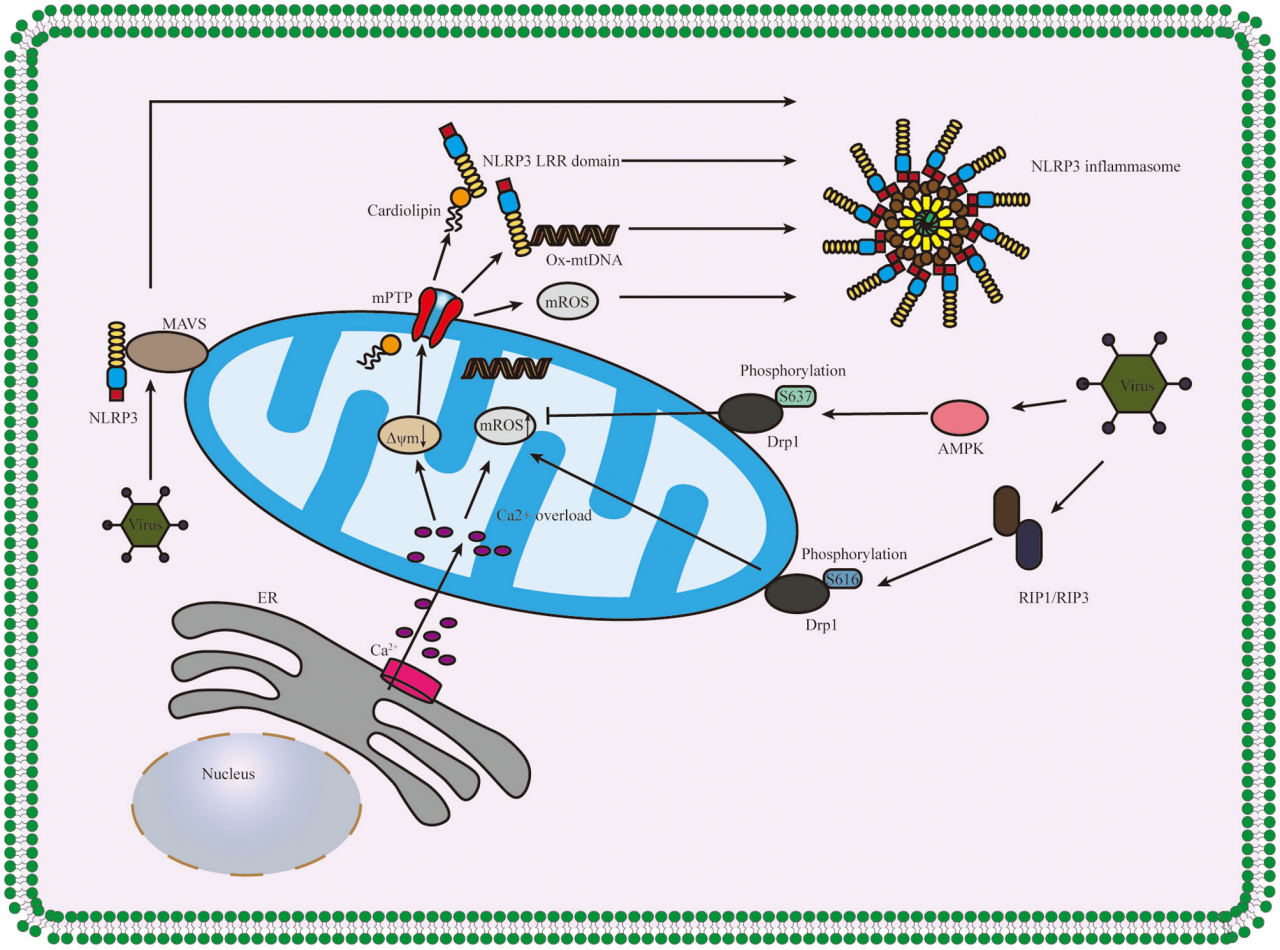
Diagram of the role of the mitochondrion in NLRP3 inflammasome activation. Ca^2+^ which released from ER influxes into mitochondria, and the overload of Ca^2+^ contributes to the high mitochondrial membrane potential (Δψm) depolarizes and the opening of mPTP, thus releasing mitochondria-derived molecules such as mROS, mtDNA and cardiolipin which produced during mitochondrial destabilization. The cardiolipin and ox-mtDNA release into cytosol and then bind to NLRP3, which might serve as NLRP3 ligands to activate the NLRP3 inflammasome. The infection of the virus accelerates the recruitment of NLRP3 to mitochondria and binds between NLRP3 and MAVS and regulates the mitochondrial destabilization and NLRP3 inflammasome activation in Drp1 phosphorylation-dependent ways. NLRP3, pyrin domain-containing protein 3; ER, endoplasmic reticulum; mPTP, mitochondrial permeability transition pore; MAVS, mitochondrial antiviral signaling protein; AMPK, AMP-activated protein kinase; RIP1/RIP3, receptor-interacting serine/threonine kinase 1 and RIP3; Δψm, mitochondrial membrane potential.

After beingstimulated, the products derived from mitochondria and other mitochondrial signaling molecules contribute to the NLRP3 inflammasome activation, such as mitochondrial ROS (mROS), mitochondrial DNA (mtDNA), and cardiolipin (Gong et al., 2018b; Zhong et al., 2018; Dagvadorj et al., 2021). All these details are shown in Figure 3. It is well-agreed that inducing ROS is the common characteristic of most NLRP3 activators (Hornung and Latz, 2010), and the main source of cellular ROS is the mitochondria (Dan Dunn et al., 2015). mROS is critical for NLRP3 inflammasome activation (Tschopp and Schroder, 2010; Gurung et al., 2015; Minutoli et al., 2016; Yu and Lee, 2016). The overexpression of mROS would activate the NLRP3 inflammasome through mainly two-signal models, namely, NF-κB pathway and mitochondria/thioredoxin-interacting protein (TXNIP), which activates the NLRP3 inflammasome (Sho and Xu, 2019). Accumulating studies have shown that the inhibitors of mtDNA synthesis and mROS can alleviate diseases by suppressing NLRP3 inflammasome activation (Zhong et al., 2016; Guo et al., 2017; Lee et al., 2019). Cardiolipin could activate the NLRP3 inflammasome in a ROS-independent way during mitochondrial destabilization. In addition to mROS, mtDNA and cardiolipin also serve as the ultimate NLRP3 ligand for the activation of NLRP3 inflammasome (Shimada et al., 2012; Iyer et al., 2013).

What is more, mitochondrial antiviral signaling protein (MAVS) is a critical regulator in the recruitment of NLRP3 to mitochondria, promoting the production of IL-1β and the pathophysiological activity of the NLRP3 inflammasome (Subramanian et al., 2013). Park et al. further discovered that MAVS not only accelerated the recruitment of NLRP3 to the mitochondria and brought it close to mtROS to improve its activation but also was involved in the assembly of NLRP3 inflammasome (Park et al., 2013). During mitochondrial destabilization, the recruitment of dynamin-related protein 1 (Drp1) on mitochondria leads to excessive mitochondrial fission, ultimately activating the NLRP3 inflammasome. It was conducted that AMP-activated protein kinase (AMPK) activation inhibited mitochondrial fission by upregulating Drp1 phosphorylation at serine637 (Ser637) in an AMPK-dependent manner to protect mitochondrial integrity and then, suppressed ER stress to inhibit the activation of NLRP3 inflammasome (Li et al., 2015, 2016; Guo et al., 2018b). What is more, it was also shown that receptor-interacting serine/threonine kinase (RIP) 1/RIP3 (RIP1-RIP3) complex could phosphorylate Drp1 at serine616 (Ser616) to activate and promote Drp1 translocation to mitochondria and ultimately contributed to the activation of NLRP3 inflammasome *via* (RIP1–RIP3)—Drp1 pathway (Wang et al., 2014). It has also proven that RIP3 regulates potassium efflux-dependent NLRP3 inflammasome activation *via* mixed-lineage kinase domain-like protein (MLKL)-induced pores and can be inhibited by supplementing extracellular potassium (Conos et al., 2017).

### Lysosomal Damage

Pyrin domain-containing protein 3 activators induce lysosomal damage, which is indirectly sensed by the NLRP3 inflammasome (Hornung and Latz, 2010). Several reports attribute the activation of the NLRP3 inflammasome to the leakage of lysosomal contents into the cytosol following phagocytosis of particulate stimuli that could damage their integrity (Hornung et al., 2008; Shimada et al., 2012). Lysosomal rupture can release cathepsins and ROS, which also significantly impacted mitochondria membrane integrity and lead to membrane permeabilization (Shimada et al., 2012). Leu-Leu-O-methyl ester (LLME) is a lysosome-damaging compound when LLME is transported to the lysosome, resulting in lysosomal membrane permeability (LMP) (Hornung and Latz, 2010). It is proved that low-dose LLME causes mild LMP and strongly activates the inflammasome (Schilling, 2016). What is more, lysosomes are emerging as intracellular Ca^2+^ stores (Zhong et al., 2017), and the activity of lysosomal ion channels and transporters maintains concentration gradients of K^+^, Ca^2+^, Na^+^, and Cl^−^ across the lysosomal membrane. Once the lysosomal damage occurs, it would contribute to the Ca^2+^ overloading and the disorder of lysosomal ion channel activity, which stimulates the activation of the NLRP3 inflammasome (Kendall and Holian, 2021). It has been proven that the restoration of lysosomal dysfunction could augment neuroprotection against ischemic stroke in neurons (Zhang et al., 2022).

## NLRP3 Inflammasome in Cerebral I/R Injury

**Figure 4:**
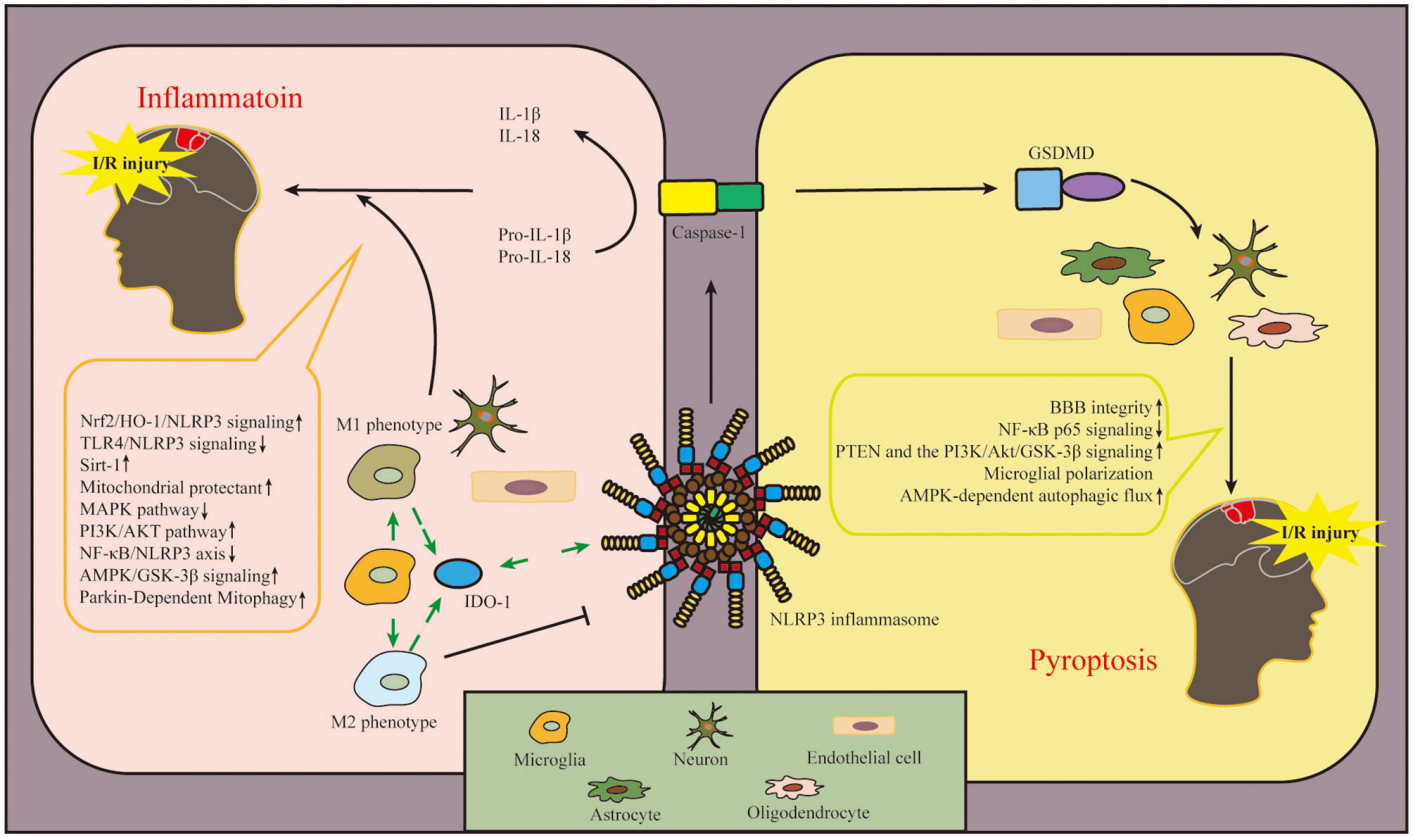
Diagram of NLRP3 inflammasome-related mechanisms after cerebral I/R injury. The involvement of NLRP3 inflammasome in the pathogenesis of cerebral I/R injury mainly includes two aspects: inflammation and pyroptosis. NLRP3 inflammasome is expressed in microglia, neuron, and endothelial cell to regulate inflammation after cerebral I/R injury. In addition, NLRP3 inflammasome is expressed in astrocytes, oligodendrocytes, microglia, neuron and endothelial cell involved with pyroptosis in ischemic tissue. In this figure, we also conclude the pathways in this review, which regulate the activation and inhibition of NLRP3 inflammasome in cerebral I/R injury. The upward arrow represents the suppression of NLRP3 inflammasome, and the downward arrow represents the activation of NLRP3 inflammasome. NLRP3, pyrin domain-containing protein 3; I/R, ischemia–reperfusion.

The main pathogenesis of ischemic stroke includes inflammation, oxidative stress, and programmed cell death (PCD) (Jin et al., 2010; Ren et al., 2021). With the involvement of the innate immune system, the activation and expression site of NLRP3 inflammasome act as a critical role in the development of ischemic stroke. In this study, we explained in detail the involvement of NLRP3 inflammasome in the pathogenesis of cerebral I/R injury, and the details are shown in Figure 4.

### Inflammation

After cerebral I/R injury, various cellular responses are aroused, such as the activation of inflammatory cytokines and the accumulation of ROS and other oxygen free radicals (Fann et al., 2013; Guo et al., 2016), which exacerbate I/R injury by promoting brain oxidative stress, inflammation, and cerebral infarction volume (Jiang et al., 2019; Franke et al., 2021; Joaquim et al., 2021). The activation of the NLRP3 inflammasome plays an important role in the development of inflammation after cerebral I/R injury. NLRP3 inflammasome is first expressed in microglia and then in microvascular endothelial cells and neurons, but finally mainly in neurons at 24 h (Gong et al., 2018b).

#### Microglia

After ischemic I/R injury, the circulating macrophages were recruited to the ischemic tissue to be involved in cerebral I/R injury (Cai et al., 2018). As brain resident macrophages, microglia could be activated first after I/R injury, and macrophages/microglia change their M1 or M2 phenotype depending on the microenvironment of the central nervous system (CNS) (Hanisch and Kettenmann, 2007; Dong et al., 2021). M1 phenotype recognizes harmful stimuli and consequently generates inflammatory cytokines such as IL-1β, IL-6, and tumor necrosis factor-α (TNF-α) (Cherry et al., 2014). M2 phenotype shifts into an anti-inflammatory state where extracellular matrix deposition, debris clearance, and angiogenesis are promoted (Varin and Gordon, 2009). Regulating the polarization of macrophage/microglia could alleviate brain damage after ischemic stroke (Ye et al., 2019). Thirty minutes after modeling MCAO in mice, activated microglia were detected in the ischemic lesions (Rupalla et al., 1998). After ischemic stroke, DAMPs and PAMPs such as ischemia, hypoxia, and inflammatory factors could activate microglia (Gülke et al., 2018) and facilitate the proinflammatory role *via* hypoxia-inducible factor 1α (HIF-1α) in microglia (Yang et al., 2014). Additionally, anti-inflammatory factors, such as IL-10 and IL-4, could induce the M2 phenotype of microglia to protect against ischemic stroke (Xiong et al., 2015). As the monitoring of the microenvironment, immune system response after ischemic stroke can also affect the polarization of microglia, such as interferon regulatory factor (IRF) 4/5 signaling (Al Mamun et al., 2018). IRF5 is required for the M1 phenotype in microglia, and IRF4 was identified as a key transcription factor for M2 polarization in microglia (Al Mamun et al., 2018). In the mouse model of MCAO, the inhibition of NLRP3 inflammasome activation in activated microglia significantly improved functional neurological deficits (Sapkota and Choi, 2021). Additionally, the polarization of M2 microglia could protect against cerebral ischemic injury *via* the NF-E2–related factor 2 (Nrf2)/heme oxygenase-1 (HO-1)/NLRP3 pathway (Wang et al., 2021c). Therefore, it is therapeutic in ischemic stroke to inhibit inflammatory response *via* inhibiting phenotype switch of microglia and NLRP3 inflammasome activation.

Indoleamine 2,3-dioxygenase 1 (IDO-1) is an immunosuppressive metabolic enzyme and elicits neuroprotective effects on ischemic injury (Park et al., 2020). IDO-1 is mainly expressed in the macrophage/microglia of the perivascular but not the parenchymal microglia of the brain (Ji R. et al., 2021). As the downstream enzyme of the NLRP3 inflammasome, the inhibition of IDO-1 with curcumin decreased NLRP3 expression (Zhang W.-Y et al., 2019); in contrast, the inhibition of IDO-1 reduced NLRP3 expression, and inhibiting NLRP3 also increased IDO-1 expression, indicating that the relationship between NLRP3 and IDO-1 is bidirectional, and the direction depends on the activation status of macrophages/microglia (Ji R. et al., 2021). It is indicated that IDO-1 may decrease the expression of NLRP3 in macrophage/microglia of the perivascular space to inhibit inflammation and protect the integrity of the blood-brain barrier (BBB) against cerebral I/R injury.

#### Microvascular Endothelial Cell

As the important structure of BBB, microvascular endothelial cells play a vital role in cerebral I/R injury. With regard to I/R inflammation, the adhesion of neutrophils to vascular endothelial cells is fundamental to the development of I/R inflammation (Dong et al., 2019). It has been studied that endothelial cells can secrete inflammatory factors such as vascular cell adhesion molecule-1 (VCAM-1) to recruit neutrophils and/or lymphocytes, leading to the infiltration of inflammatory cells in the ischemic region (Gao et al., 2021). Lysophosphatidylcholine (LPC) is the main active component of oxidized low-density lipoproteins (ox-LDLs), and it has been proven that LPC could facilitate inflammatory response in brain microvascular endothelial cells (BMECs) *via* G protein-coupled receptor 4 (GPR4)-mediated activation of NLRP3 inflammasomes (Liu et al., 2021a). The expression inhibition of NLRP3 inflammasome in endothelial cells could improve the integrity of BBB and behavioral outcomes (Cao et al., 2016). The functional integrity of BMECs has shown a significant protective effect against brain I/R injury (Wang et al., 2021a).

#### Neuron

After the onset of cerebral I/R injury, cellular damage is mainly triggered by excitotoxicity, mitochondrial disturbances, dysfunction of the endoplasmic reticulum, ROS production, calcium toxicity, nitric oxide toxicity, zinc toxicity, and PCD (Lo et al., 2005; Hossmann, 2006). After the ischemic injury, neuron death occurs within minutes (Amantea et al., 2009), and the neuronal cell death after cerebral I/R injury included apoptosis, autophagy, pyroptosis, ferroptosis, parthanatos, phagoptosis, and necroptosis (Tuo et al., 2022). With the time prolongation, the neuron cell and nuclear membrane in the center of the ischemic region ruptured, and the cells were lysed (Gou et al., 2021). As mentioned earlier, the cellular damage could contribute to the activation of the NLRP3 inflammasome, ultimately exacerbating the inflammatory response. Based on the fact that NLRP3 inflammasome is ultimately mainly expressed in neurons after ischemic stroke, drugs that inhibit NLRP3 inflammasome to alleviate cerebral I/R injury and reduce inflammatory response must be studied.

### Pyroptosis

Pyroptosis is one kind of pro-inflammatory PCD, which has been shown to be involved in cerebral I/R injury (Voet et al., 2019). Different from other PCD, pyroptosis is characterized by the rupture of the plasma membrane to form pores, increases in cell permeability, and the release of inflammatory cytokines (Fink and Cookson, 2005). The occurrence of pyroptosis relies on caspase-1/4/5/11, and caspase-1 and caspase-11 are the main activated inflammatory caspases during cerebral ischemia (Gou et al., 2021). The oxidative stress and inflammatory response activate the NLRP3 inflammasome and consequently induce the cleave of pro-caspase-1 to form active caspase-1, which ultimately promotes pyroptosis *via* the canonical inflammasome pathway (Broz, 2015). Caspase-1 cleaves pro-IL-1β and pro-IL-18 into a mature form of IL-1β and IL-18. Caspase-1 could also cleave Gasdermin D (GSDMD), expose Asp280 amino acid sites, and promote the recruitment of the N-domain of GSDMD to the cell membrane, leading to the cell membrane pore formation and pyroptosis (Zhang et al., 2019; Ji et al., 2021), thereby releasing IL-1β and IL-18, and leading to cascade inflammatory response and inflammatory cell recruitment (Vande Walle and Lamkanfi, 2016).

Pyroptosis has been shown to be expressed in various CNS cells, such as microglia, neuron, oligodendrocytes, and astrocytes (McKenzie et al., 2018; Zhou et al., 2019; Hu et al., 2021; Zhao et al., 2021). Additionally, it has been proven that the pyroptosis of endothelial cells participates in the pathogenesis of cerebral I/R injury (Wang et al., 2021d). Regarding the critical downstream effector of pyroptosis (Shi et al., 2015), the expression of GSDMD was found to increase after I/R and peak at 3–5 days in mice (Lu et al., 2021) and so aggravates I/R-induced cerebral infarction and brain injury. The ablation of GSDMD exerts a neuroprotective effect by inhibiting microglia pyroptosis in mice after cerebral I/R injury (Wang et al., 2020a). Moreover, the inhibition of NLRP3 inflammasome-dependent pyroptosis could reduce neuronal injury and cerebral infarct after I/R injury (Kang et al., 2021; Shi et al., 2022). Regarding the upstream of pyroptosis, the suppression of NLRP3 inflammasome is a critical target for the treatment of cerebral I/R injury.

## Therapeutic Approaches Targeting NLRP3 Inflammasome for Cerebral I/R Injury

As a neurological disease with a high disability rate, ischemic stroke still lacks efficient therapeutic treatment in the clinic. As previously mentioned, NLRP3 inflammasome plays a major role in cerebral I/R injury, and many drugs that inhibit NLRP3 inflammasome activation have been studied for the treatment of ischemic stroke. In this study, we summarized the therapeutic approaches involving NLRP3 inflammasome and classified these drugs as follows: clinical treatment, herbal/natural component, and novel inhibitor.

### Novel Inhibitor

To find out the potential pathogenesis and therapeutic drugs for cerebral I/R injury application, many novel inhibitors have been found to mitigate cerebral I/R injury by suppressing the activation of the NLRP3 inflammasome. The most well-studied inhibitor is MCC950, which is proven specificity in inhibiting NLRP3 inflammasome targeting for the assembly of NLRP3 inflammasome (Wu et al., 2020). MCC950 can modify the active conformation of NLRP3, and it prevents NLRP3 oligomerization in response to external stimulation (Tapia-Abellán et al., 2019). Experimental studies have found that the treatment of MCC950 could reduce the infarction and edema and improved neurological deficits and BBB integrity *via* inhibiting inflammatory cytokines, pyroptosis, and brain oxidative stress in the ischemic region after MCAO (Ismael et al., 2018; Bellut et al., 2021; Joaquim et al., 2021). CY-09 could inhibit ATPase activity and block NLRP3 oligomerization to inhibit the activation of NLRP3 inflammasome (Jiang et al., 2017). It has shown the therapeutic effect of CY-09 in cerebral I/R injury *via* inhibiting NLRP3 inflammasome-induced inflammation and pyroptosis (Sun et al., 2020; Franke et al., 2021). Oridonin could prevent NLRP3 inflammasome complex assembly against NLRP3 inflammasome activation (He et al., 2018). The study found oridonin prevented oxidative stress-induced endothelial injury *via* promoting the Nrf2 pathway and thereby repaired BBB integrity, alleviated neuroinflammation, and infarct volume after ischemic stroke (Li et al., 2021a).

### Clinical Treatment

Although the pathogenesis of ischemic stroke remains unclear, many conventional medicines revealed the therapeutic effect on ischemic stroke function by inhibiting the activation of NLRP3 inflammasome. As an oxygen radical scavenger, Edaravone reduced neurotoxicity, oxidative stress, and inflammatory response after cerebral I/R injury (Xu et al., 2021a). Indobufen and Aspirin and their Combinations with Clopidogrel or Ticagrelor (IACT) could alleviate pyroptosis *via* NF-κB/NLRP3 pathway after cerebral I/R injury, which indicated that the combination of antiplatelet drugs is a promising strategy for the curation of cerebral I/R injury (Li et al., 2021c). In addition, idebenone, as a mitochondrial protectant, was found to decrease ROS and cytosolic oxidized mtDNA, suppress uncontrolled NLRP3 activation, and consequently alleviate cerebral inflammatory response after cerebral I/R injury (Peng et al., 2020). Hypothermia is used for clinical neuroprotective purposes after ischemic stroke. It was suggested that hypothermia downregulated the expression of NLRP3 and attenuated I/R-induced pyroptosis partially *via* the phosphatidylinositol-3-kinase (PI3K)/Akt/Glycogen synthase kinase-3β (GSK-3β) pathway (Diao et al., 2020). Electroacupuncture (EA) is commonly used to relieve chronic pain and stroke rehabilitation, and some studies have uncovered that EA pretreatment protects against transient cerebral I/R injury (Wang et al., 2012). EA stimulus has a neuroprotective effect through α7nAChR by modulating the inhibition of NLRP3 inflammasome-associated inflammatory response and cellular apoptosis (Jiang et al., 2019).

### Herbal/Natural Component

Herbs andtheir extracts have been an important source of approach for the treatment of ischemic stroke, many herbal medicines have shown neuroprotection after cerebral I/R injury *via* inhibiting NLRP3 inflammasome activation, and the detailed information is summarized in Table 2. *Tongxinluo* (TXL) is a common Chinese patent drug used clinically in the treatment of stroke, and TXL could protect ischemic brain tissues against pyroptosis in astrocytic by inactivating caspase-11/GSDMD (Wang et al., 2021b). *Xingnaojing* injection (XNJ) is isolated from famous traditional Chinese medicine prescriptions named *An-Gong-Niu-Huang Wan*, which is well-accepted in the clinic due to its significant therapeutic effect (Lai et al., 2017). *XNJ* ameliorated neurological deficits and BBB disruption following I/R injury in a manner of NLRP3 inflammasome suppression (Qu et al., 2019). The effective compounds extracted from *Buyang Huanwu Decoction* (BYHWD) can alleviate neuronal damage and inhibit NLRP3 inflammasome-mediated neuronal pyroptosis (She et al., 2019). Hispidulin is a component widely existing in traditional Chinese medicine and could inhibit I/R-induced pyroptosis in the ischemic cortex by modulating AMPK/GSK3β signaling (An et al., 2019). Resveratrol, anthocyanin, melodinhenine B, and 6-Gingerol could alleviate cerebral I/R injury by inhibiting NLRP3 inflammasome activation (He et al., 2017; Cui et al., 2018; Li et al., 2020a; Luo et al., 2021). Icariin, a flavonol glycoside extracted from *Epimedium brevicornum* Maxim (Berberidaceae), has shown an anti-inflammatory effect against Oxygen and Glucose Deprivation/Reoxygenation (OGD/R) through the inositol-requiring enzyme-1 (IRE1)/X-box binding protein 1 (XBP1) pathway in microglia (Mo et al., 2021). Moreover, the pretreatment of sulforaphane (SFN) in the ischemic stroke model could protect neurovascular and alleviate neurological deficits and BBB disruption *via* the Nrf2/HO-1 defense pathway (Alfieri et al., 2013; Warpsinski et al., 2020). Herbal medicines especially traditional Chinese medicines have been an essential approach for the recovery of ischemic stroke, identifying natural herbs which ameliorate I/R injury *via* NLRP3 inflammasome that has prospective value.

**Table 2:** Herbal drugs that target NLRP3 inflammasome after cerebral ischemia–reperfusion (I/R) injury.

**Drugs**	**Type**	**Source**	**Target**	**Signaling**	**Animals and cells**	**Models**	**Current clinical trial**	**References**
Icariin (ICA)	Glycoside	Epimedium brevicornum Maxim (Berberidaceae)	NLRP3 and caspase-1	IRE1/XBP1s pathway	Microglia	Oxygen-glucose deprivation (OGD/R)	–	Mo et al., 2021
*Tongxinluo*	TCM prescription	Jiang Xiang, Ru Xiang, Bing Pian, Ren Shen, Chi Sao, Suan Zao Ren, Tan Xiang, Can Tui, Shui Zhi, Tu Bie Chong, Quan Xie, Wu Gong	Inhibited Astrocytic Pyroptosis	Caspase-11/GSDMD	Sprague–Dawley rats	Middle cerebral artery occlusion/ reperfusion (MCAO/R)	Cardiocere brovascular diseases of blood stasis syndrome	Wang et al., 2021b
6-Gingerol	Phenolic compound	Ginger	NLRP3 and caspase-1	TRPV1/FAF1 complex	Sprague–Dawley rats	MCAO	–	Luo et al., 2021
Bakuchiol (BAK)	Prenylated phenolic mono-terpene	the seeds of psoralea corylifolia	NLRP3 and caspase-1	Nrf2 signaling	Mice and BV-2 cells	MCAO and OGD/R	–	Xu et al., 2021b
Gastrodin (GAS)	Versatile compound	Traditional Chinese herb Tianma	NLRP3 and caspase-1	LncRNA NEAT1/miR-22-3p/ NLRP3	Sprague–Dawley rats	MCAO	–	Zhang et al., 2021
Oridonin (Ori)	Diterpenoid isolated	Rabdosia rubescens	NLRP3 and caspase-1	NF-κB signaling	C57BL/6 mice and BV-2 cells	MCAO and OGD/R	–	Jia et al., 2021
D-Carvone	D- carvone dietary monoterpenes	Seed variety caraway essential oil	NRLP3	TLR4/NLRP3 signaling pathway	Sprague–Dawley rats	MCAO	–	Dai et al., 2020
Cepharanthine (CEP)	Bibenzyliso quinoline (BBI) alka-loids	Stephania cepharantha	NRLP3	12/15-LOX signaling	Mice and BV-2 cells	MCAO and OGD/R	–	Zhao et al., 2020
Tetrandrine	Alkaloid	Radix Stephania tetrandra	NRLP3	Sirt-1	Mice	MCAO	–	Wang et al., 2020b
Astilbin	Dihydroflavonol derivative	Rhizoma Smilacis glabrae (RSG)	NRLP3	MAPK pathway and PI3K/AKT pathway	PC12 cell	OGD/R	–	Li et al., 2020b
Melodinhenine B	Eburnean-vindolinine-type bisindole alkaloid	M. henryi	NRLP3	BBB integrity	Sprague–Dawley rats	MCAO	–	Li et al., 2020a
*XingNaoJing*	TCM prescription named An-Gong-Niu-Huang pill	Moschus, Radix Curcumae, borneol and Fructus gardeniae	NLRP3	BBB integrity	Sprague–Dawley rats	MCAO	the treatment of stroke	Qu et al., 2019
Glycosides	Astragaloside IV, paeoniflorin, and amygdalin	Buyang Huanwu Decoction	NLRP3	Classical pyroptosis pathway	Sprague–Dawley rats	MCAO	Prevent and treat cerebral ischemia	She et al., 2019
Hispidulin	Flavonoid	Chinese herbal medicines	NLRP3	AMPK/GSK3β signaling pathway	Sprague–Dawley rats and primary cerebral astrocytes	MCAO and OGD/R	–	An et al., 2019
Anthocyanin	Phenolics or polyphenolics	*Myrica rubra*	NLRP3	TLR4/NF-κB and NLRP3 Pathways	ICR mice	MCAO	–	Cui et al., 2018
Resveratrol (RSV)	Poly-phenolic compound	*Veratrum grandiflorum*	NLRP3	Sirt1-dependent autophagy induction	Sprague–Dawley rats	MCAO	–	He et al., 2017
Sulforaphane (SFN)	Isothiocyanate	Cruciferous vegetables	NLRP3	The activation of NLRP3 inflammasome	Sprague–Dawley rats	MCAO	–	Yu et al., 2017

### Others

Intriguingly, non-coding RNA (ncRNA), including long non-coding RNA and microRNA, is involved in the pyroptosis, inflammatory response, oxidative stress, apoptosis, and BBB permeability in a manner of NLRP3 inflammasome after cerebral I/R injury (Ghafouri-Fard et al., 2020), and all the information is shown in Table 3. It is of great importance to develop drugs that inhibit NLRP3 by targeting ncRNAs.

**Table 3:** Selected noncoding RNAs involving NLRP3 inflammasome in cerebral I/R injury.

**ncRNA**	**TUG1**	**NEAT1**	**MiR-139**	**MiR-668**
Target	miR-200a-3p	miR-22-3p	c-Jun	Mitochondrial function
Expression	Upregulation	Upregulation	Downregulation	Upregulation
Species	Adult C57BL/6 mice	Male Sprague–Dawley rats	Human neuroblastoma cells and mouse microglia cells	Male Sprague-Dawley rats
Model	MCAO	MCAO	OGD/R	MCAO
Treatment	Knockdown of TET2	Gastrodin	MiR-139 mimics	MiR-668 inhibitor
Pathway	TUG1/miR-200a-3p/NLRP3	NEAT1/miR-22-3p Axis	c-Jun/NLRP3 inflammasome	MiR-668/NLRP3
Therapeutic effect	Attenuate I/R-induced inflammatory response and brain injuries	Improve the neurological scores of rats, reduce the area of cerebral infarction, and inhibit pyroptosis	Inhibit NLRP3 inflammasome-mediated pyroptosis and inflammatory response	Modulate mitochondrial function and regulate NLRP3 signaling
	Yin et al., 2021	Zhang et al., 2021	Wang Q.-S et al., 2020	He and Zhang, 2020
References				

Bone marrow mesenchymal stem cell-derived exosomes (BMSC-Exos) can attenuate the activation of NLRP3 inflammasome and NLRP3 inflammasome-mediated pyroptosis *via* promoting AMPK-dependent autophagic flux in OGD/R injury (Zeng et al., 2020), by a mechanism that switches microglial phenotypes from M1 to M2, so as to ameliorate cerebral I/R injury (Liu et al., 2021b). Moreover, intermittent fasting can attenuate the inflammation and neuronal damage following cerebral I/R injury through the suppression of NLRP3 inflammasome activation (Fann et al., 2014). Additionally, an enriched environment (EE) could rescue neurological deficits after I/R injury *via* inhibiting the activities of NLRP3 inflammasome and attenuating neuronal pyroptosis (Liu et al., 2021c).

The continuous studies for NLRP3 inflammasome will promote the new drug research and development for cerebral I/R injury clinical treatment, but further research is needed for the clinical application of new compounds.

## Discussion

Despite efforts to understand the pathophysiology of ischemic stroke, no effective neuroprotective drugs have been identified to modulate brain damage following ischemic stroke in human due to the complexity of ischemic stroke. With a fall of cerebral blood flow in ischemic stroke, the ischemic region contains two aspects, namely, ischemic core and penumbra, the damage to the brain in the penumbra is reversible based on the ionic homeostasis and transmembrane electrical potentials (Astrup et al., 1981), and how to restore blood flow in penumbra is a therapeutic target for the ischemic stroke clinic treatment (Ramos-Cabrer et al., 2011). Due to the sudden and rapid onset, ischemic stroke is often treated out of the therapeutic time window and, therefore, produces irreversible neuronal death (Sommer, 2017). As the most well-characterized inflammasome, NLRP3 inflammasome is closely related to the inflammatory response and pyroptosis; therefore, the blocking of NLRP3 inflammasome becomes a significant therapeutic target for ischemic stroke. Therefore, it is meaningful to downregulate the expression of NLRP3 inflammasome after cerebral I/R injury. The abnormal expression of NLRP3 inflammasome is not only detected in lab experiments but also is exactly confirmed in burgeoning clinical evidence (Chen et al., 2013; Zheng et al., 2013). It is shown that the neuronal upregulation of NLRP3 is an early event within the first 24 h of cerebral I/R injury which corresponds to the hyperacute and acute phase of human stroke (Franke et al., 2021). After the onset of ischemic stroke, the inhibition of NLRP3 inflammasome according to its expression time sequence could be considered as a future therapeutic target. Within 24 h of the occurrence of ischemic stroke, the early activation of NLRP3 inflammasome in microglia and subsequent activation in neurons should be effectively targeted according to the cell type, thereby, reducing cerebral I/R injury (Chumboatong et al., 2022; Wang et al., 2022), but the exact time needs further experimental studies. The NLRP3 inflammasome is a critical part of the innate immune system which regulates the cleaves and secretion of proinflammatory cytokines, such as IL-1β and IL-18 in response to the DAMP and PAMP signaling. Inflammatory cytokines are involved with the secondary brain injury in cerebral I/R, especially IL-1β and IL-18. It is influential for the treatment of cerebral I/R to regulate the expression of inflammatory cytokines under control, thus the involvement with inflammatory response makes NLRP3 inflammasome extensively investigated for assembly and activation. In addition to inflammation, the activation of NLRP3 inflammasome in ischemic brain tissue promotes the activities of pyroptosis and eventually aggravates brain injury. According to the activation and assembly of the NLRP3 inflammasome, molecules and compounds interfering with NLRP3 activation can alleviate cerebral I/R injury by downregulating the expression of the NLRP3 inflammasome in animal experiments. Importantly, these inhibitors have not been conducted in clinical trials due to their limited pharmacokinetic profiles and safety. Moreover, it is proven that the impact of the unspecific medications (SFN, Genipin) on the inflammasomes besides NLRP3 is negligible in the treatment of ischemic stroke (Franke et al., 2021). Therefore, highly specific and efficient inhibitors that focus on inhibiting the NLRP3 inflammasome and can effectively permeate the cell membrane and BBB will be an important topic for their research and clinical application.

Interestingly, it is a hot spot where mitochondria are closely related to NLRP3 inflammasome activation. Mitochondria are the central hub in innate and adaptive immune cells (Breda et al., 2019), and at the same time, mitochondria also participate in pyroptosis, a kind of cell death, which is related to cerebral I/R injury (Gurung et al., 2015). NLRP3 inflammasome triggers caspase-1-dependent mitochondrial damage. Caspase-1 activates multiple pathways to precipitate mitochondrial disassembly, which leads to the mROS production and dissipation of mitochondrial membrane potential, mitochondrial permeabilization, and mitochondrial network fragmentation (Yu et al., 2014). The molecules derived from mitochondria such as mROS, mtDNA, and cardiolipin are important regulators for the activation of the inflammasome, and mtDNA and cardiolipin are found to bind to NLRP3 and serve as a ligand for the activation of NLRP3 inflammasome. Now that the proven studies conducted that mitochondria are essential for NLRP3 inflammasome activation, the further studies are kindly suggested to focus on the specific mechanism by which mitochondria regulate the activation of NLRP3 inflammasomes.

## Conclusion and Future Perspective

The role of NLRP3 inflammasome in cerebral I/R injury is mainly concentrated on NLRP3 inflammasome-dependent cytokine release and pyroptosis, which makes NLRP3 inflammasome a target therapeutic protein in cerebral I/R injury. Although the essential of NLRP3 inflammasome activation in ischemic stroke has been proved, its specific role needs to be further explored. It is expected that the more effective pharmacokinetic profiles and safe medicines for NLRP3 inflammasome could be used for cerebral I/R injury treatments in clinical.

## Author Contributions

This study is finished under the guidance of SY. LW and WR proposed the idea of this manuscript. LW wrote the original draft. WR critically edited and revised the manuscript. QW, HX, and TL are responsible for the editing. YW, JD, and CZ investigated the literature. All authors contributed to the article and approved the submitted version.

## Funding

This study was supported by the Science and Technology Department of Sichuan Province (Grant No. 2019YFS0543); Sichuan Administration of Traditional Chinese Medicine (2020ZD002, 2021ZD015, and 2020JC0150); the strategic cooperation project between Luzhou Municipal People's Government (2019LZXNYDC02); China Postdoctoral Science Foundation (Grant No. 2020M683365); and the National Natural Science Foundation of China (Grant No. 82074378).

## Conflict of Interest

The authors declare that the research was conducted in the absence of any commercial or financial relationships that could be construed as a potential conflict of interest.

## Publisher's Note

All claims expressed in this article are solely those of the authors and do not necessarily represent those of their affiliated organizations, or those of the publisher, the editors and the reviewers. Any product that may be evaluated in this article, or claim that may be made by its manufacturer, is not guaranteed or endorsed by the publisher.
